# Early and progressive deficit of neuronal activity patterns in a model of local amyloid pathology in mouse prefrontal cortex

**DOI:** 10.18632/aging.101136

**Published:** 2016-12-20

**Authors:** Fani Koukouli, Marie Rooy, Uwe Maskos

**Affiliations:** ^1^ Institut Pasteur, Département de Neuroscience, Unité Neurobiologie intégrative des systèmes cholinergiques, 75724 Paris Cedex 15, France; CNRS, UMR 3571, Paris, France; ^2^ Group for Neural Theory, Laboratoire de Neurosciences Cognitives, INSERM Unité 969, Département d’Études Cognitives, École Normale Supérieure, Paris, France

**Keywords:** Alzheimer’, s Disease, AAV, human mutated APP, chronic two-photon imaging, neuronal synchronicity

## Abstract

Alzheimer's Disease (AD) is the most common form of dementia. The condition predominantly affects the cerebral cortex and hippocampus and is characterized by the spread of amyloid plaques and neurofibrillary tangles (NFTs). But soluble amyloid-β (Aβ) oligomers have also been identified to accumulate in the brains of AD patients and correlate with cognitive dysfunction more than the extent of plaque deposition. Here, we developed an adeno-associated viral vector expressing the human mutated amyloid precursor protein (AAV-hAPP). Intracranial injection of the AAV into the prefrontal cortex (PFC) allowed the induction of AD-like deficits in adult mice, thereby modelling human pathology. AAV-hAPP expression caused accumulation of Aβ oligomers, microglial activation, astrocytosis and the gradual formation of amyloid plaques and NFTs. *In vivo* two-photon imaging revealed an increase in neuronal activity, a dysfunction characteristic of the pathology, already during the accumulation of soluble oligomers. Importantly, we found that Aβ disrupts the synchronous spontaneous activity of neurons in PFC that, as in humans, is characterized by ultraslow fluctuation patterns. Our work allowed us to track brain activity changes during disease progression and provides new insight into the early deficits of synchronous ongoing brain activity, the “default network”, in the presence of Aβ peptide.

## INTRODUCTION

Alzheimer's Disease (AD) is a devastating neuro-degenerative condition that greatly impacts society, primarily affecting the elderly population, and will become an enormous burden as the population ages [1]. In the broad category of dementia, AD amounts to 70% of all cases and thereby is the most common form of dementia [2]. Although there are familial genetic mutations linked to AD, the sporadic form is the most prevalent. There is currently no cure available for AD, only symptomatic treatments are administered in order to slow down the progression of the clinical manifestations [3].

Several genes have been implicated in AD in humans, most notably, those encoding the mutated APP, presenilin 1 and presenilin 2 [4]. Consequently, various transgenic mouse models of AD harboring mutations in these genes have been established to decipher the disease mechanism. Moreover, a significant advance in the field came from the development of transgenic rodent models that exhibit tau pathology [5,6]. Although the results from these models have unraveled specific components of the disease pathology and given indications for developing potential therapies, there is no transgenic model that replicates the broad spectrum of AD pathology. In some cases, the mutations are associated with other pathologies, like frontotemporal dementia that is not part of AD pathology [7]. Moreover, in transgenic mice that express human familial AD mutations, the gene is expressed throughout the brain, making it impossible to study disease induced changes in specific brain regions.

The two primary features associated with AD pathology are the senile plaques and the neurofibrillary lesions. Since deposited Aβ in amyloid plaques had a lack of correlation with cognitive impairment and its location and quantity in the brain, the 'toxic Aβ oligomer' hypothesis developed as a possible alternative mechanism [8]. Aβ oligomers are associated with AD hallmarks, like inducing abnormal tau phosphorylation [9]. Soluble and highly toxic forms of Aβ such as oligomers and protofibrils may be more directly linked to cellular pathology, however the balance between monomeric Aβ, oligomers and insoluble Aβ fibrils is poorly understood and the lack of an experimental description of the toxic Aβ oligomer makes conclusions difficult [8]. In addition, the acute short-term injection of Aβ oligomers, sometimes of defined composition, into the brain of rodents and non-human primates [10] does not allow studies on the progression of the disease over time. There still is a need for a model that replicates the regionality and timecourse of the disorder.

Here, we developed an adeno-associated virus (AAV) based model for replicating AD-like pathology. The AAV vector expresses the mutated form of human APP harboring three pathogenic mutations: Swedish, London and Austrian (hAPP-SLA) [11–13]. We chose these mutations because they are associated with early onset of the disease. We targeted the expression of hAPP- SLA to the PFC, due to this region's key role in cognitive processes and its crucial implication in AD pathology [14] and we recorded neuronal spontaneous activity patterns. Our findings reveal a distinct impact of Aβ oligomers in the synchronous activity of the PFC neurons and provide a mechanistic basis for understanding the pathophysiology of the disorder.

## RESULTS

### Generation of an AAV vector to express human mutated hAPP

**Figure 1:**
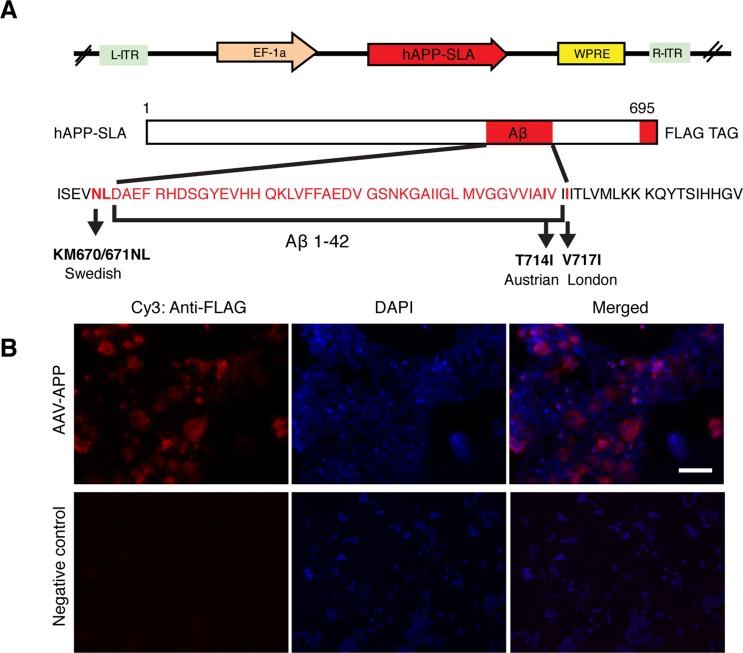
Production of an AAV vector to express human mutated APP (**A**) Adeno-associated virus construct encoding for the 695 amino acid isoform of the human APP harbouring three pathogenic mutations (hAPP-SLA). The mutations in the APP sequence are highlighted. S: Swedish, L: London, A: Austrian mutations. (**B**) A HEK cell line was transfected with the viral vector expressing APP-SLA. Cells were positive for APP (red) and visualized with the anti-FLAG antibody. Negative control: HEK cells without being transduced with the viral vector and stained with the anti-FLAG antibody. Scale bar = 50 μm.

The mutant hAPP-SLA contained the ***S***wedish, ***L***ondon and ***A***ustrian mutations. The Swedish mutation was chosen because it leads to familial AD, as seen in patients harboring just one allele of this dominant mutation [11]. It has been widely used to establish transgenic mouse models of AD, for example the Tg2576 line [15], and in combination with other fAD associated mutations, such as PS1 (M146V) and tau (P301L) in the 3x-Tg mouse model [16]. The Austrian (T714I) [12] and London (V717I) [13] mutations were added, that when present in hAPP are known to drive a higher production of Aβ. In addition, we a FLAG tag at the C- terminus of the hAPP sequence to be able to detect the protein with anti-FLAG tag antibodies. We thus produced an AAV-hAPP-SLA-FLAG construct (Fig. 1A).

Protein expression was confirmed *in vitro* in the HEK cell line. HEK cells were transduced with the vector expressing hAPP-SLA. The anti-FLAG antibody confirmed the synthesis of the hAPP protein (Fig. 1B).

### *In vivo* detection of hAPP in the PFC

**Figure 2:**
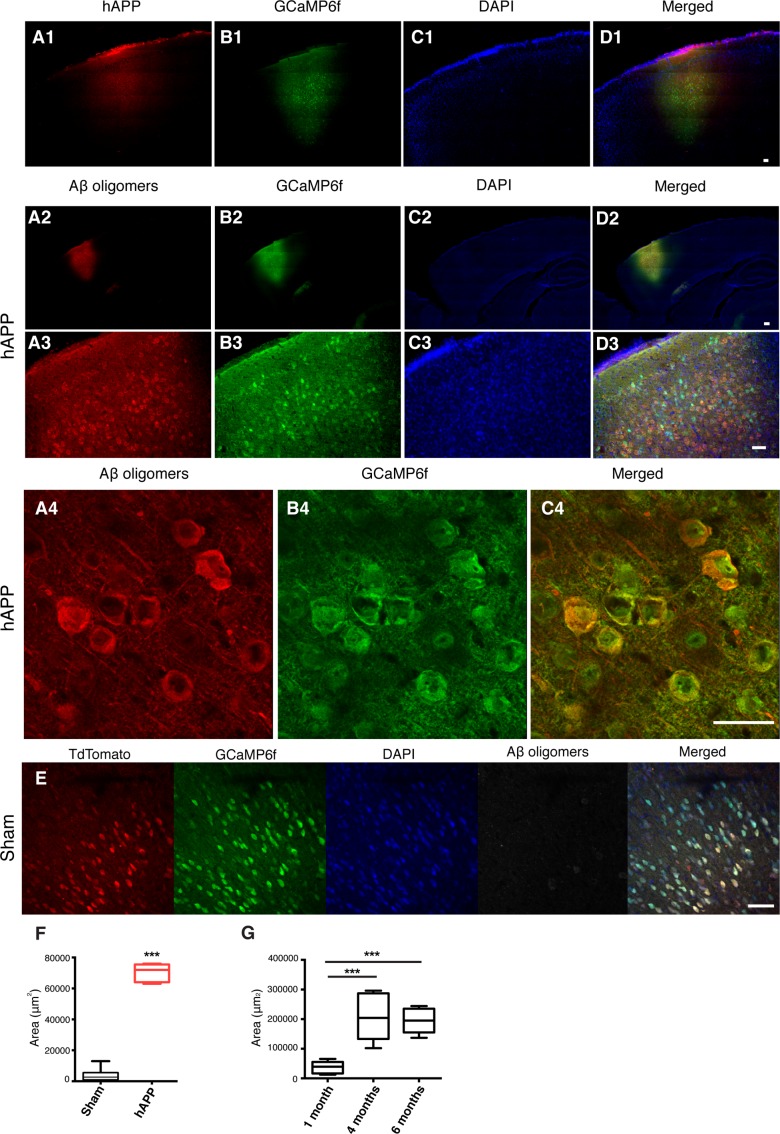
Detection of hAPP and Aβ oligomers in the PFC at 1 mpi of AAV-hAPP-SLA in WT mice *In vivo* detection of hAPP (**A1**), Aβ oligomers (**A2-A4**), GCaMP6f (**B**), DAPI (**C**) and merged (**D**). Immunofluorescence images at low (1, 2), medium (3) and high (4) magnification. Scale bars = (1) 100 μm, (2, 3) 200 μm and (4) 50 μm. (**E**) Aβ oligomers were not detected in sham mice injected with the control vector AAV-tdTomato. Scale bar = 50 μm. (**F**) Quantification of Aβ oligomer diffusion in sham and hAPP mice. (**G**) Quantification of Aβ oligomer diffusion in hAPP mice at three different timepoints. (Student's test, P < 0.0001, 3 mice for each group).

The main cellular pathology of AD consists of damage and loss of neurons in widespread areas of the cortex and hippocampus [17]. The cognitive impairments characteristic of dementia in humans, such as attentional deficits and short-term memory loss, indicate PFC pathology [18, 19]. For this reason, we targeted the prelimbic area (PrL) of the PFC. A series of stereotaxic injections were performed *in vivo* to verify the efficiency of the AAV-hAPP-SLA in the PrL cortex (PrLC) of 3 month old WT mice. The vector showed diffusion in the brain and sufficient expression of the transgene, as visualized one month post-injection using an anti-FLAG antibody (Fig. 2 panel 1) with the hAPP diffusing throughout the PFC. As shown in the mosaics, there is no labeling in other parts of the brain outside PFC (Fig. 2 panel 1).

### AAV-hAPP-SLA drives Aβ oligomer synthesis and intracellular accumulation

Most AD transgenic models exhibit memory impairments, with the cognitive deficits occuring earlier than the appearance of extracellular plaques. Research shifted to identify the precursors to plaque formation and to determine whether, and how, aggregation of Aβ was crucial to its toxicity. This led to the focus on soluble oligomeric Aβ species. As in AD transgenic mouse models, cognitive decline in humans is not proportional to Aβ plaque load [20], but does correlate with soluble Aβ species [21]. Intraneuronal Aβ has gained experimental support in recent years, as, similar to humans, many hAPP AD transgenic mice exhibit intraneuronal amyloid accumulation [22]. The accumulation of intracellular Aβ has been shown to precede deposition. Interestingly, it was found that intraneuronal Aβ strongly correlates with initial deficits on a hippocampal-based memory task [23] and that intraneuronal Aβ is more neurotoxic than extracellular Aβ [24].

We investigated whether the expression of the hAPP- SLA protein was able to drive Aβ oligomer accumulation in our model. The presence of oligomeric Aβ was confirmed with anti-VHH 31-1 antibody, specific for oligomeric forms of Aβ [25]. Immunofluorescent images with this antibody in WT mice injected with AAV-hAPP-SLA showed abundant intracellular Aβ oligomer expression in the PFC at one month post-injection (1 mpi) of viral vector (Fig. 2, panels 2 to 3). Aβ synthesis was strictly related to hAPP-SLA expression, since Aβ oligomers were not observed in other brain areas, without viral transduction. There was no detection of Aβ oligomers in sham mice injected with the control vector AAV-CAG-tdTomato (Fig. 2E, F). We followed the Aβ oligomer accumulation over time and performed immunostaining analysis in mouse brains at various time points. Significant Aβ production was also detected 4 and 6 mpi of AAV-hAPP-SLA. The diffusion of Aβ was significantly higher 4 and 6 mpi in comparison to 1 mpi of the AAV-hAPP-SLA (Fig. 2G).

### Activation of microglia by AAV-hAPP-SLA

Neurological disorders trigger local inflammation and consequently activation of the immune response. Specifically, AD is characterized by an inflammatory response to Aβ, including the activation of microglia and the recruitment of astrocytes around Aβ deposits [26]. Cause or consequence of this activation in the disease progression is still not clear. Several studies in animal models suggest that microglia activation precedes amyloid plaques [27, 28] and the formation of NFTs [29, 30]. Once activated, microglia prominently change their morphology: the ramified processes swell and withdraw, while their cell bodies enlarge [31, 32]. Microglia activation occurs in the AD brain with microglia clusters forming around amyloid deposits as an early indicator of pathology and little is known about how this interaction is initiated. Here, we specifically focused on the early stages of the pathology and we validated our AD model by characterizing *in vivo* the process by which the microglia become activated in the presence of Aβ peptide.

**Figure 3:**
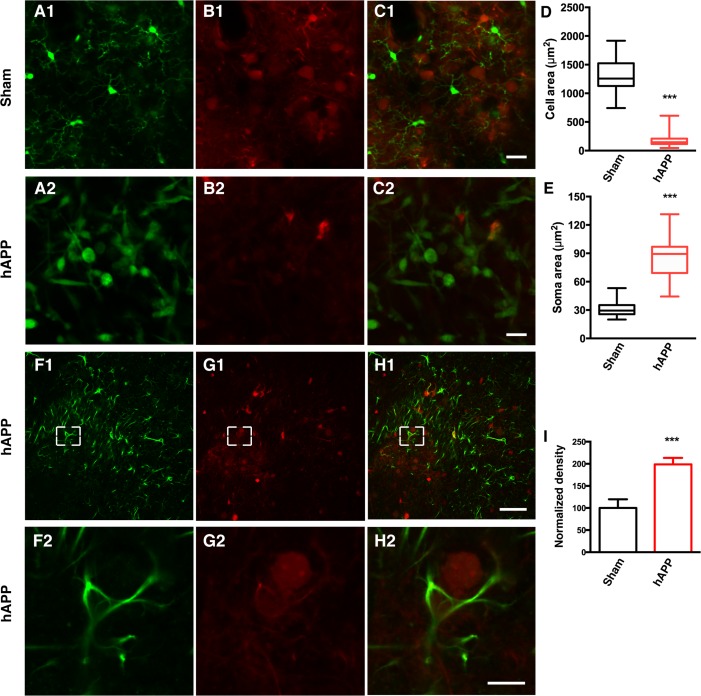
AAV-hAPP-FLAG induces microglial activation and astrocytosis *In vivo* two-photon imaging of microglial activation in AAV-hAPP-SLA injected CX3CR1-GFP^+/−^ mice. (**A1-A2**), GFP expressing microglial cells, (**B1-B2**), tdTomato and (**C1-C2**), merged. (1) Sham-operated mice indicating microglia cells in resting state, (2) AAV-hAPP-SLA injected mice indicating microglia cells in an activated state. Mean cell area (**D**) and mean soma area of microglia cells (**E**) in the sham and AAV-hAPP-SLA injected mice (Student's test, P < 0.0001, n = 150 cells, 3 mice). (**F-H**) Representative images showing GFAP immunostaining in the PFC. Immunofluorescence images at low (1) and high (2) magnification for GFAP (**F**), Aβ oligomers (**G**) and merged (**H**). (**I**) Quantification of GFAP density. The density was normalized to control levels. The error bar is ± SEM. (Student's test, P < 0.001, 6 slices were analyzed from 3 control mice and 6 slices from 3 AAV-hAPP-SLA injected mice). Scale bars = 50 μm, for H2 scale bar = 10 μm.

CX3CR1-GFP^+/−^ mice [33] were used to visualize microglia in order to characterize the morphological dynamics of microglia activation. The knockin of GFP at the Cx3cr1 locus in CX3CR1-GFP mice results in GFP-labeling of microglia. CX3CR1-GFP^+/−^ mice were injected with a mixture of AAV-hAPP-SLA and the AAV1.CAG.tdTomato, whereas control mice were injected only with the AAV1.CAG.tdTomato vector specifically in the PrLC. A cranial window was implanted (see *Materials and Methods* for details) and four weeks after the injection, the microglia were imaged by two-photon microscopy with the mouse lightly anesthetized with isoflurane (0.8% isoflurane /O_2_). Ten-second interval imaging of control mice showed microglia that were characterized by a small cell body and highly elaborated thin processes, with multiple branches extending radially, a feature of the resting state (Fig. 3A1 and Supplementary Movies 1 and 2). Interestingly, in the mice injected with the AAV-hAPP-SLA vector, microglia were characterized by an amoeboid form, a feature of microglial activation (Fig. 3A2 and Supplementary Movie 3). Their processes were retracted and the soma enlarged. Most of the activated microglia had a large round morphology with one short or complete lack of processes. We estimated the level of microglial extension by measuring the cell area, including the soma and the processes and we found a significant difference between the two groups. The mean cell area of microglial cells in the sham mice was 1335 ± 50.21 μm^2^, and 172.9 ± 13.66 μm^2^ for the AAV-hAPP-SLA mice (Student's test, P < 0.0001, n = 150 cells, 3 mice) (Fig. 3D). The mean soma area of microglial cells in the sham mice was 30.66 ± 1.269 μm^2^, and 87.35 ± 3.623 μm^2^ for the AAV-hAPP-SLA mice (Student's test, P < 0.0001, n = 150 cells, 3 mice) (Fig. 3E). Thus, the AAV-hAPP-SLA induces robust microglial activation.

### AAV-hAPP-SLA induces astrocyte activation

Reactive gliosis, including astrocyte activation, detected by increased glial fibrillary acidic protein (GFAP) expression, is another important characteristic of AD neuropathology [34]. Therefore, we aimed to investigate whether our model induces astrocyte activation. Sections from the PFC of control or AAV-hAPP-SLA injected mice were immunostained for the presence of astrocytes using an anti-GFAP antibody. The AAV-hAPP-SLA injected mice showed markedly increased immunoreactivity for GFAP, compared to the control (Fig. 3F to I). This suggests that the presence of astrocyte-mediated inflammatory processes is associated with the Aβ oligomers.

### Amyloid plaque and neurofibrillary tangle formation in AAV-hAPP-SLA injected mice

**Figure 4:**
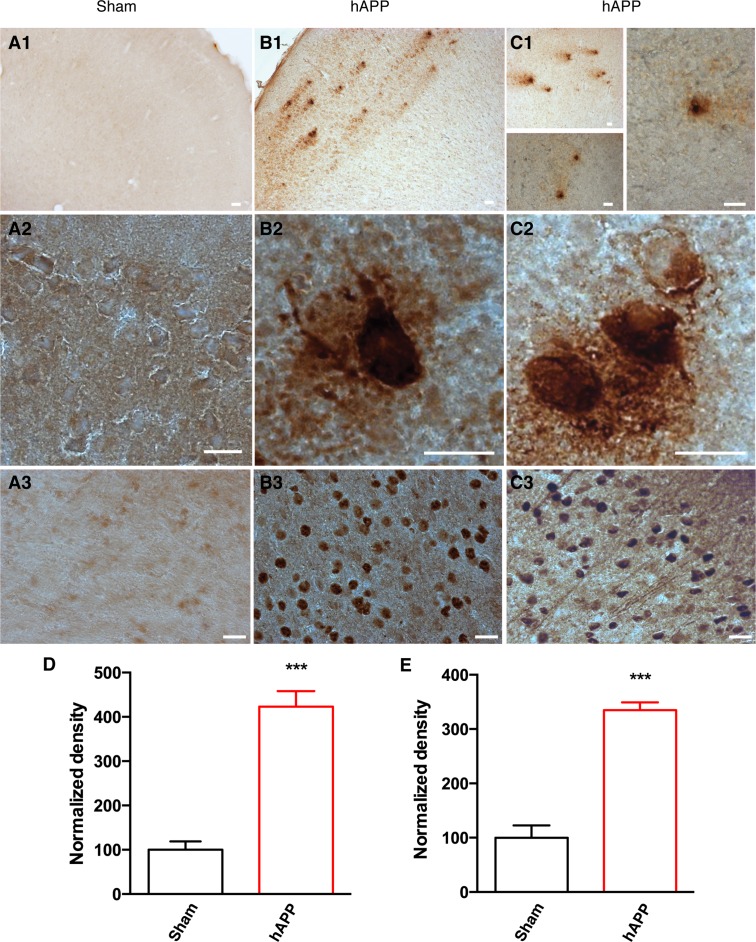
Detection of amyloid plaques and NFTs in the PFC of AAV-hAPP-SLA injected mice at 12 mpi (**A**) Sham mouse brains injected with a control AAV and (**B**), (**C**) AAV-hAPP-SLA injected mice stained with the 4G8 antibody (1, 2) and AT100 antibody (phospho-tau at Ser212 and Thr214) (3). Scale bars = 20 μm. Quantification of 4G8 optical density (**D**) and AT100 densities (**E**) and normalized to values obtained in sham operated mice. The error bar is ± SEM. (Student's test, P < 0.001, for the quantification of amyloid plaques, 8 slices were analyzed from 4 control mice and 6 slices from 3 AAV-hAPP injected mice, whereas for the quantification of NFTs, 6 slices were analyzed from 3 control mice and 6 slices from 3 AAV-hAPP injected mice).

As the disease advances, amyloid peptides accumulate and aggregate, eventually forming amyloid plaques. We were able to detect typical amyloid plaques in the PFC of AAV-hAPP-SLA mice at 12 mpi, but not in control mice (Fig. 4). Another hallmark pathology of human AD is the intra-neuronal aggregation of hyper-phosphorylated tau forming NFTs. We aimed to evaluate abnormal tau phosphorylation in our model. We investigated the levels of paired helical filaments (PHFs) reactive to the anti-AT100 antibody, which recognizes tau phosphorylated at serine 212 and threonine 214 residues [10]. AT100-positive neurons represent early stage markers of tau pathology. Immunostaining with AT100 revealed tau pathology in the PFC of mice injected with the AAV-hAPP-SLA, but no immunoreactivity was observed to the PFC of control mice (Fig. 4). No amyloid plaques or tau pathology were detected before 12 mpi.

The amyloid cascade hypothesis predicts that tau hyperphosphorylation occurs as a downstream consequence of Aβ accumulation [35]. APP-over-expressing transgenic mice have provided evidence both for and against this. Unlike humans with AD, many mouse models do not develop NFTs, yet many do show increased tau hyperphosphorylation.

### Higher rates of neuronal activity in layer II/III of PrLC in AAV-hAPP-SLA injected mice

There is accumulating experimental evidence that neuronal hyperactivity as a result of amyloid pathology is a major indicator of AD associated dysfunction [36].

Experimental analysis using various approaches, from single neurons to neuronal populations to large-scale networks, with a variety of electrophysiological and imaging techniques, have revealed two forms of AD-related hyperactivity and provided first insights into the synaptic mechanisms. A striking early observation from *in vivo* two-photon calcium imaging in mouse models of AD was the unexpected abundance of hyperactive neurons in networks of the cerebral cortex and the hippocampus. For instance, in the frontal cortex of amyloid plaque-bearing hAPP and PS1 double transgenic mice (the APP23 PS45 model), more than 20% of supragranular layer II/III neurons were found to be hyperactive [37]. These hyperactive neurons were located mostly in the direct vicinity of amyloid plaques, less than 60 μm from the plaque border, whereas the fractions of the simultaneously present functionally silent neurons increased with plaque distance.

**Figure 5:**
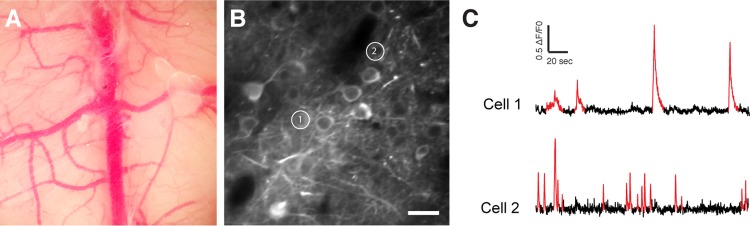
*In vivo* two-photon calcium imaging (**A**) Chronic cranial window. (**B**) Two-photon image of GCaMP6f expressing neurons. (**C**) Spontaneous Ca^2+^ transients (in red) of the two cells shown in (B). Scale bar = 50 μm.

**Figure 6:**
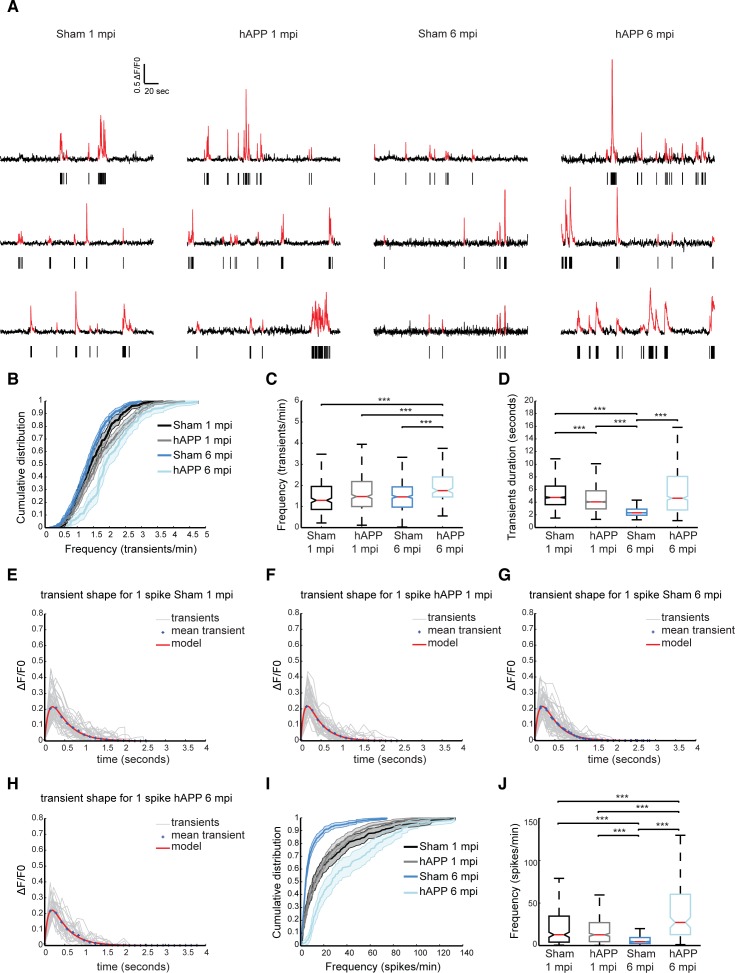
Higher rates of neuronal activity in layer II/III of PrLC in AAV-hAPP-SLA injected mice (**A**) Representative spontaneous Ca^2+^ transients of sham-operated mice and hAPP mice and corresponding raster plots 1 and 6 mpi. (**B**) Population averaged cumulative plot of the distribution of spontaneous transients/min. (**C**) Median frequency of spontaneous calcium transients/min of the different mouse groups. (**D**) Median transient durations. (**E-H**) Kernel estimation for the different mouse groups. The kernel (model) is obtained from the fitting of an alpha function on the mean unitary calcium transient (crosses). In light gray, individual unitary calcium transients used to compute the mean for sham-operated and hAPP mice at 1 and 6 mpi of the vector. (**I**) Population averaged cumulative histograms of the distribution of spontaneous spikes/min. (**J**) Median frequency of spontaneous calcium spikes/min of the different mouse groups (n = 4 animals for each group). Kruskal-Wallis test for all comparisons. (*P<0.05, **P<0.01 and ***P<0.001)

In order to assess neuron function in the PFC of our AD mouse model, we used dynamic two-photon microscopy to image the activity of neurons *in vivo* through a chronic imaging window (Fig. 5A). Neurons of PrLC were transduced with an AAV expressing the genetically encoded calcium indicator GCaMP6f driven by the synapsin promoter [38]. Four weeks after AAV injection, the majority of layer II/III neurons exhibited green fluorescence (Fig.5B). The activity patterns of lightly anesthetized mice, 0.8% isoflurane/O2, were recorded. Three-month-old WT mice injected with the AAV-hAPP-SLA (hAPP) and sham mice, injected with the control vector (see *Materials and Methods*), were used to monitor the spontaneously occurring somatic Ca^2+^ transients in individual cells (Fig. 5C). We analyzed the spontaneous neuronal activity at two time points: 1 and 6 mpi (Fig. 6A).

The distribution of spontaneous Ca^2+^ transients for the different time points is shown in Figure 6B. We found that the median frequency distribution of Ca^2+^ transients shifted towards higher values in hAPP mice at 6 mpi (1.75 ± 0.08 transients/min, 160 cells, n = 4 mice), compared with the sham (1.458 ± 0.04 transients/min, 829 cells, n = 4 mice, Kruskall Wallis test; P < 0.001), although at 1 mpi no significant difference was found (sham, 1.29 ± 0.05 transients/min in 451 cells; hAPP, 1.47 ± 0.049 transients/min in 729 cells, n= 4 mice in each group) (Fig. 6C). We also analyzed the duration of Ca^2+^ transients between the different mouse groups. For the sham mice the median transient duration was 4.74 ± 0.17 seconds (2542 transients) and 4.04 ± 0.15 for the hAPP mice (3960 transients analyzed, Kruskall Wallis test; P < 0.001), at 1 mpi. Interestingly, the sham mice at 6 mpi exhibited a much lower calcium transient duration when compared to the hAPP mice (sham, 2.32 ± 0.04 seconds of 5524 transients analyzed; hAPP, 4.63 ± 0.29 seconds of 887 transients analyzed n = 4 mice, Kruskall Wallis test; P < 0.001) (Fig. 5D).

Ca^2+^ transients were then selected according to their shape. Unitary calcium transients detected with GCaMP6f should have a rapid rise period, followed by a single peak value and a longer decay period [38]. We assumed that the smallest and fastest Ca^2+^ were a result of a single action potential. The mean shape and amplitude of this unitary event was used as a kernel for deconvolution to best estimate spike frequency. This procedure was performed on all recorded transients separately for each experimental condition, i.e., Sham and hAPP, 1 and 6 mpi (Fig. 6E to H and Table 1). We found layer II/III neuronal activity significantly increased in PrLC of hAPP mice (27.14 ± 3.33 spikes/min) compared to WT-control mice (4.46 ± 0.71 spikes/min, Kruskall Wallis test; P < 0.001), 6 mpi. There was no significant difference between the mice imaged at 1 mpi (WT control; 12.70 ± 2.35 spikes/min, hAPP; 12.65 ± 1.38 spikes/min, p = 0.397 Kruskall Wallis test; Fig. 6I, J). Interestingly, sham mice showed a decrease in neuronal activity over time.

**Table 1:** Kernel estimation of amplitude, tau rise and tau decay of PFC layer II/III neurons in the different mouse groups

Mouse group	Amplitude (ΔF/F) (%)	tau rise (msec)	tau decay (msec)
Sham 1 mpi	21.5202 ± 0.576	200 ± 5	390 ± 10
hAPP 1 mpi	21.7686 ± 0.4782	160 ± 1	350 ± 1
Sham 6 mpi	21.5057 ± 0.35565	190 ± 1	370 ± 5
hAPP 6 mpi	22.2633 ± 0.99118	180 ± 1	370 ± 10

Data presented as mean ± SEM.

### Neuronal synchronicity is disrupted early in the disease

**Figure 7:**
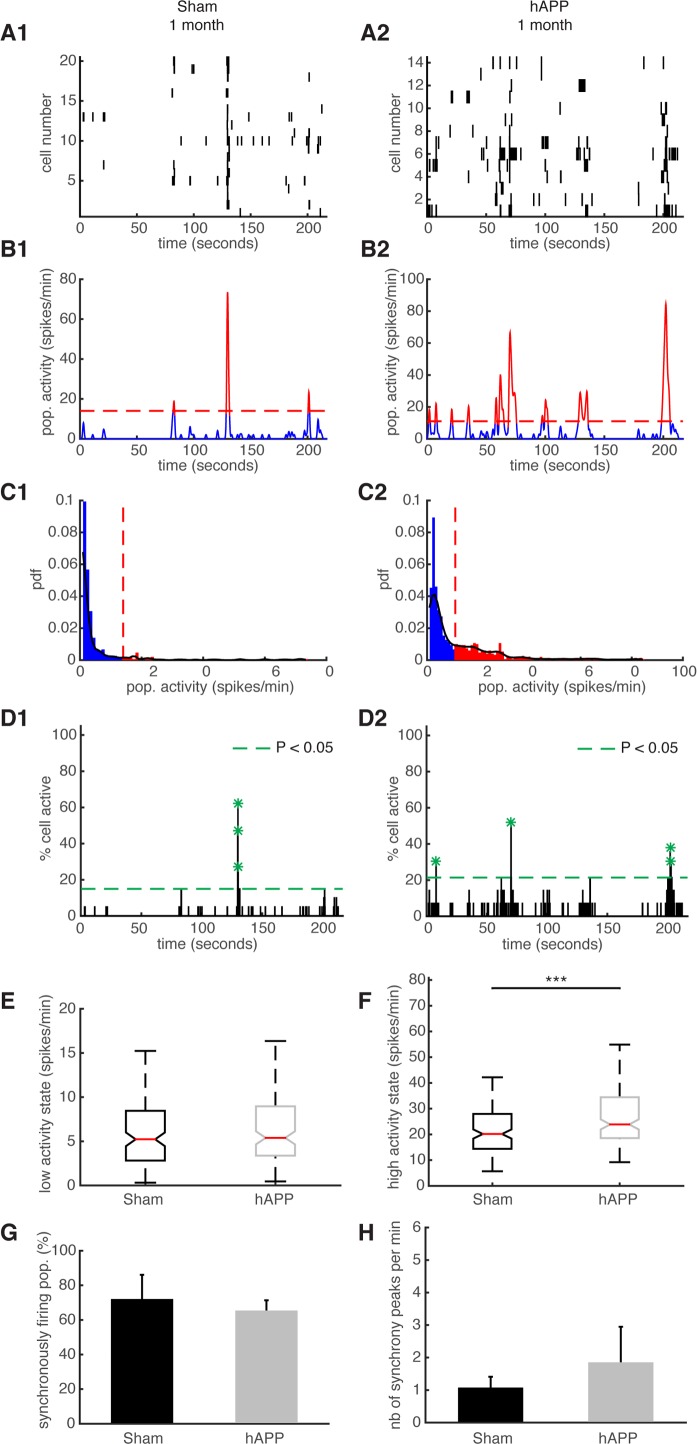
Synchronous firing of simultaneously recorded neurons in sham and hAPP mice 1 mpi (**A**) Representative raster plots for one population of simultaneously recorded neurons. Each row corresponds to the spiking activity of one neuron. (**B**) Mean neural activity for the populations in A. The activity was smoothed through Gaussian filtering. The red periods correspond to the high activity states and the blue periods to the low activity states. The dotted red line represents the computed threshold between the two states. (**C**) Probability density function (pdf) of the population activity exhibited in (B). The black line represents the smoothed pdf, through Gaussian filtering. Red bars represent the high activity states and blue bars the low activity states. (**D**) Histogram representing the percentage of cells active in small time bins (∼ 0.144 sec), for the population activity in (A). Asterisks mark significant peaks of synchrony. (**E**) Boxplots of low activity states for each mouse group (spikes/min). (**F**) Boxplots of high activity states for each mouse group (spikes/min). (**G**) Percentage of populations (simultaneously imaged neurons) exhibiting synchronous activity. (**H**) Mean number of synchrony peaks per minute for the different animal groups. (1) sham and (2) hAPP mice.

We have previously shown that the ongoing activity in the mouse PFC constantly fluctuates and exhibits synchronously firing neuronal activity, similar to humans [39]. These ultraslow fluctuations are considered to be related to elementary physiological processes associated with conscious processing in humans [39,40]. We aimed to identify ultraslow fluctuations in hAPP mice and compare their properties with the fluctuations observed in age-matched sham mice. Representative examples of simultaneously recorded neurons are shown in Fig. 7A. To identify patterns of activity characterized by high/low activity state transitions, we studied the distribution of their time varying mean activity, as before [39]. The high activity states correspond to population activities in red and low activity states correspond to population activities in blue (Fig. 7B and C). The activity patterns were then analyzed in order to detect synchronous activity in populations of simultaneously recorded neurons (Fig. 7D). We found that 66.25 ± 6.88 % of simultaneously recorded populations exhibited high/low activity states in sham mice and 73.064 ± 13.67 % in hAPP mice 1 mpi with no significant difference between groups (P = 0.65, ANOVA). In each population of simultaneously recorded neurons with high/low activity transitions, we determined the percentage of cells that exhibit an activity pattern in accordance with the population activity. In sham mice, 73.91 ± 8.67 % of cells fire in accordance with their population activity and in hAPP, 73.68 ± 5.41 % with no significant difference between the groups (P = 0.83, Kruskal-Wallis test). In addition, we found that 45.96 ± 10.63 % of simultaneously recorded cells exhibited high/low activity states in sham mice and 52.23 ± 13.31 % in hAPP mice 1 mpi with no significant difference (P = 0.73, ANOVA). Next, we computed the high/low activity properties in all populations. The low activity duration was 5.2 ± 2.26 s for sham mice and 6.5 ± 2.9 s for hAPP mice 1 mpi (P = 0.26, Kruskal-Wallis), whereas the high activity state duration was 3.32 ± 1.08 s for sham mice and 3.6 ± 1.1 s for hAPP mice 1 mpi, with no significant difference (P = 0.28). The high activity state for hAPP mice was significantly higher (23.88 ± 1.96 spikes/min) than for sham mice 1 mpi (20.16 ± 3.04 spikes/min, P < 0.001) whereas there was no significant difference for the low activity state (sham: 5.22 ± 2.09 spikes/min, hAPP: 5.4 ± 0.55 spikes/min; P = 0.74, Kruskal-Wallis) (Fig. 7E and F).

We then analyzed and compared the synchronicity in the two groups 1 mpi. In the sham mice, neurons displayed synchronous activity in 72.12 ± 14% of the recorded populations, whereas in hAPP mice neurons displayed synchronous activity in 65.4 ± 5.9% with no significant difference between groups (P = 0.37, ANOVA) (Fig. 7G). The number of synchrony peaks detected was similar between sham mice (1.27 ± 0.34 peaks/min) and hAPP (2.08 ± 1.07 peaks/min, P = 0.54, ANOVA) (Fig. 7H). Also, the percentage of coactive cells in the peaks of synchrony was similar between sham (50.66 ± 2.21 %) and hAPP mice (51.86 ± 1.23 %, P = 0.61), 1 mpi.

**Figure 8:**
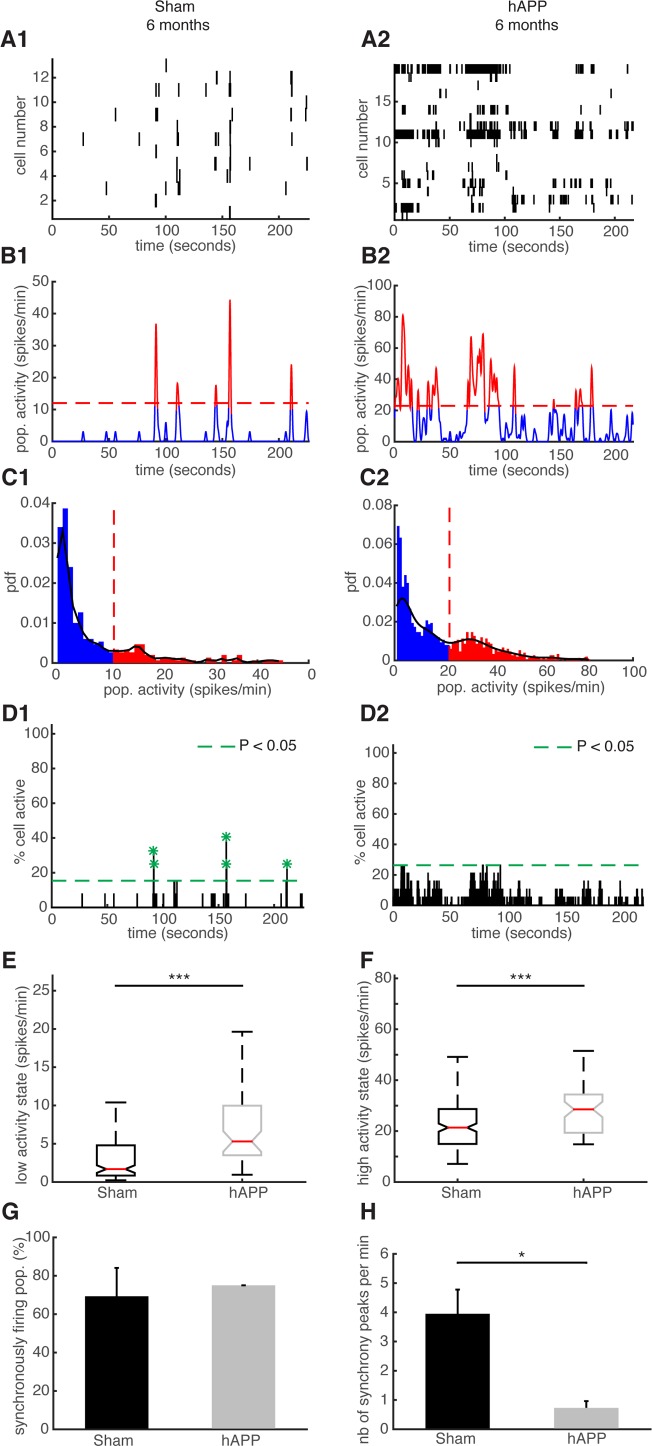
Neuronal synchronicity is disrupted in the PFC of hAPP mice 6 mpi **(A)** Representative raster plots for one population of simultaneously recorded neurons. Each row corresponds to the spiking activity of one neuron. (**B**) Mean neural activity for the populations in (A). The activity was smoothed through Gaussian filtering. The red periods correspond to the high activity states and the blue periods to the low activity states. The dotted red line represents the computed threshold between the two states. (**C**) Probability density function (pdf) of the population activity exhibited in (B). The black line represents the smoothed pdf, through Gaussian filtering. Red bars represent the high activity states and blue bars the low activity states. (**D**) Histogram representing the percentage of cells active in small time bins (∼ 0.144 sec), for the population activity in (A). Asterisks mark significant peaks of synchrony. (**E**) Boxplots of low activity states for each mouse group (spikes/min). **(F**) Boxplots of high activity states for each mouse group (spikes/min). (**G**) Percentage of populations (simultaneously imaged neurons) exhibiting synchronous activity. (**H**) Mean number of synchrony peaks per minute for the different animal groups. (1) sham and (2) hAPP mice.

We then performed the same type of analysis for the same mice at 6 mpi. Representative examples of simultaneously recorded neurons are shown in Fig. 8A. The high activity states correspond to population activities in red and low activity states correspond to population activities in blue (Fig. 8B and C). Synchronous activity in populations of simultaneously recorded neurons was detected for both mouse groups (Fig. 8D). We determined that 79.33 ± 11.57 % of simultaneously recorded populations exhibited high/low activity states in sham mice and 87.5 ± 0.11 % in hAPP mice 6 mpi with no significant difference between groups (P = 0.78, ANOVA). In sham mice, 84.61 ± 5.35 % of cells fire in accordance with their population activity and in hAPP, 85 ± 5.2 % with no significant difference between the groups (P = 0.95, Kruskal-Wallis). Moreover, 54.61 ± 12.02 % of simultaneously recorded cells exhibited high/low activity states in sham mice and 71.59 ± 0.11 % in hAPP mice 6 mpi with no significant difference (P = 0.59, ANOVA). The low activity duration was 16.96 ± 2.74 s for sham mice and 9.96 ± 2.98 s for hAPP mice 6 mpi (P = 0.02, Kruskal-Wallis), whereas the high activity state duration was 2.26 ± 0.22 s for sham mice and 3.17 ± 0.77 s for hAPP mice 6 mpi, with a significant difference (P = 0.005). The high activity state for hAPP mice was significantly higher (28.5 ± 21.36 spikes/min) than for sham mice 6 mpi (21.37 ± 1.30, P < 0.001), but also the low activity state was significantly higher for hAPP mice than for sham mice 6 mpi (sham: 1.69 ± 0.69 spikes/min, hAPP: 5.3 ± 2.17 spikes/min; P < 0.001, Kruskal-Wallis) (Fig. 8E and F). In the sham mice, neurons displayed synchronous activity in 69.29 ± 14.79% of the recorded populations, whereas in hAPP mice neurons displayed synchronous activity in 75 ± 0.01% with no significant difference between groups (P = 0.88, ANOVA) (Fig. 8G). Importantly, the number of synchrony peaks detected was robustly decreased for the case of hAPP mice (0.72 ± 0.23 peaks/min) compared to sham mice 6 mpi (3.95 ± 0.82 peaks/min) (P = 0.049, ANOVA) (Fig. 8H). Also, the percentage of coactive cells in the peaks of synchrony was increased for hAPP mice (41.4 ± 4.83 %) compared to sham mice (31.18 ± 0.52 %, P < 0.001 ANOVA), 6 mpi.

Overall, neuronal synchronicity is disrupted in the presence of Aβ oligomers 6 mpi of the AAV-hAPP in the absence of amyloid plaques or NFTs.

## DISCUSSION

AD is a complex disease affecting discrete brain areas with defined specificity for certain regions and pathways [41]. To more effectively study AD pathology, it is necessary to dissect the pathological mechanisms using *in vivo* models and recording methods identifying the functional changes.

Here, we generated an AAV model that created AD-like pathology. The AAV was made to express mutant human APP harboring the Swedish, London and Austrian mutations that are associated with early onset of AD. Production of different AAVs for the expression of the tau protein or the mutant APP have previously been described without including any functional analysis on the role of soluble Aβ [42]. With injection of our viral vector into the PFC of WT mice, we successfully induced Aβ production as early as 1 mpi. This production of Aβ oligomers in the initial phase of the pathology has the potential to greatly advance the understanding of the mechanisms centrally implicated in the early stages of AD pathogenesis, with applications for therapeutic development. It is thought that in AD the balance between Aβ production and clearance is disrupted therefore causing an increase in cellular Aβ. Over time, this disrupted balance leads to large amyloid deposits. The deposition of Aβ is a slowly progressive process that starts in the neocortex and then expands hierarchically into other brain regions, representing different phases of Aβ deposition [43]. In accordance with the sequential involvement of brain regions in AD, we targeted the prefrontal area of the brain, a region associated with cognitive functions that could provide a basis for the understanding of the early phases of the disease. We were able to identify microglia activation, a feature of AD pathology, at 1 mpi of the vector. We followed the disease progression and identified hallmarks of AD pathology, such as the presence of amyloid plaques and tau hyper-phosphorylation that appeared one year after vector injection.

Our method could be very useful to investigate region specific vulnerability in AD linked to Aβ deposition and to follow the accumulation of amyloid deposition from one targeted brain structure to connected areas. In contrast, transgenic mouse models that express mutated hAPP globally in the brain throughout pre- and post-natal development into adulthood complicate the ability to measure the disease progression. Since all brain regions express the mutated APP, it is impossible to determine how the disease pathology spreads between an affected brain region to other initially unaffected areas. Our model allows targeted induction of AD-like pathology and therefore future studies exploring the spread to other brain regions will be possible.

Another advantage of this approach is that it is applicable to every animal model, strain or species. This includes knock-out animals for specific factors involved in the pathways that lead to AD pathology. Notably, the cholinergic pathway, and cholinergic neurons located in the basal forebrain in particular, are subject to degeneration in AD [44]. Nicotinic acetylcholine receptors are crucially implicated in cognitive processing and have been proposed to be involved in the cognitive decline observed in AD patients [45]. In human tissue, Aβ oligomers have been identified in cholinergic neurons, suggesting a role in cholinergic deficiency [46]. The AAV vector we developed and characterized *in vivo* can be used to rapidly study the implications of different nicotinic receptor knock-out animals by using a single injection and therefore avoids the time and resource consuming crossbreeding between transgenic lines.

Furthermore, our new AD model is compatible with chronic long-term *in vivo* imaging and recording techniques. The importance of this is illustrated by previous slice and primary neuronal culture studies that concluded that high levels of Aβ cause a reduction in excitatory neuronal transmission resulting in hypoactivity and synaptic failure [47]. However, clinical observations suggested that patients with AD, and in particular early-onset familial AD, have a higher incidence of epileptic seizures, indicating an increase rather than decrease in neuronal excitability [48]. *In vivo* studies in the last few years have further confirmed these human results in mouse models. It was shown that hyperactive neurons were found in close proximity to amyloid plaques, suggesting a synaptotoxic microenvironment around the AD lesions [37]. Importantly, one study, in visual cortex, demonstrated that a progressive deterioration of neuronal tuning for the orientation of visual stimuli occurs in parallel with the age-dependent increase of the Aβ load, and this deterioration was found only in neurons that are hyperactive during spontaneous activity [49]. The mechanisms underlying the changed neuronal activity in the diseased visual cortex are likely to involve a redistribution of synaptic inhibition and excitation, as it has been suggested for the impaired spontaneous activity in the frontal cortex [37]. In addition, local application of soluble Aβ oligomers in the form of synthetic dimers provoked hyperactivity of CA1 neurons in WT mice [50].

Here, we examined the alterations in PFC neuronal activity at two different timepoints. By using *in vivo* two photon imaging, chronic cranial windows and monitoring neuronal activity with a genetically encoded calcium indicator, we were able to track neuronal activity in the same mice, at 1 and 6 mpi of the AAV-hAPP-SLA vector. Interestingly, we identified a robust increase in the median frequency of PFC neurons injected with the AAV-hAPP-SLA as compared to control mice. At 6 mpi, we did not detect amyloid plaques or the formation of NFTs, however, the expression of amyloid oligomers can explain the occurrence of the increased neuronal activity. Our results are in accordance with human studies where asymptomatic humans with high amyloid load were found to display abnormally increased activation in the default-mode network using blood-oxygen-level dependent (BOLD) fMRI. This increase was mainly found in the medial prefrontal cortex, the precuneus and the posterior cingulate cortex [51]. In addition, we observed reduced neuronal activity in the PFC of control mice at 6 mpi. This finding is in accordance with neuroimaging studies in humans where older adults have shown reduced activity in PFC and other brain regions critical for cognitive functions [52]. Our data show an evolution in the functional properties of the neurons and network since the brain activity of the same mouse was followed over time. A crucial role of soluble Aβ on the spontaneous brain activity was identified, since increased neuronal activity was detected before the formation of amyloid plaques. Our findings indicate that Aβ is able to increase spontaneous neuronal activity early in the disease progression in the absence of amyloid plaques or NFTs and shed a different light on previous work that demonstrated a role of amyloid plaques in cortical hyperactivity [37].

In addition, we have previously shown that in the resting state, in the absence of any explicit task performance or external stimulus, the PFC exhibits a highly informative mode of spontaneous activity that is characterized by ultraslow fluctuations and synchronized activity patterns [39]. Here, we analyzed hAPP mice for the presence of ultraslow fluctuations and synchronously firing neurons and compared their properties with sham mice. Interestingly, high and low activity states were increased in the presence of Aβ peptide 6 mpi, however, the number of synchronous peaks was robustly decreased. Computational and experimental studies had established that a neuronal group is the most fundamental unit in the cortex and it is not formed by a single neuron, but by a cluster of tightly coupled neural cells, which fire in synchrony [53]. We have previously shown that nicotinic acetylcholine receptors (nAChRs) are specifically required for synchronized activity patterns in the mouse PFC [39]. In addition, pharmacological intervention with nicotinic antagonists is enough for the disruption of neuronal synchronicity [39]. Here, we also found that Aβ is able to disrupt neuronal synchronicity early in the disease progression in the absence of amyloid plaques or NFTs, implying a possible role of nAChRs in the Aβ-mediated disruption of synchronicity.

In conclusion, our model induces Aβ accumulation, astrocyte and microglia activation, amyloid plaque formation, and abnormal tau phosphorylation. Functional *in vivo* two-photon imaging of AAV-hAPP injected mice revealed an important role of soluble Aβ on spontaneous brain activity, indicating that the spontaneous synchronous activity patterns are disrupted in an AD-like brain before the formation of amyloid plaques. These findings can further our understanding of AD mechanisms of pathogenesis since it replicates important features of AD and can therefore be applied to improve our efforts to develop future therapies, already targeting early AD stages. However, the exact mechanisms that lead to the disruption of neuronal activity in AD is indeed poorly understood and further studies must be performed in order to elucidate the complexity of AD mediated hyperactivity and disruption of synchronicity.

## MATERIALS AND METHODS

### Adeno-associated viral construction

The generation of the hAPP-SLA-FLAG plasmid in pGEM-T was described previously [54]. The hAPP-SLA-FLAG cassette was recovered from the pGEM-T vector with XbaI and EcoRV restriction enzymes and inserted in an AAV-EF1a vector. The AAV-EF1a vector was derived from an AAV-EF1a-DIO-ChetA-EYFP plasmid (http://www.everyvector.com/sequences/show_public/7300) that was digested with the same restriction enzymes. Ligation of the two fragments with T4 DNA ligase (M0202, NEB) resulted in the adeno-associated viral vector AAV-EF1a-hAPP-SLA-FLAG. Virus production was performed by INSERM U649 Vector Core of Nantes University to a final titer of 2.2×10^12^ vg/ml.

### Animals

Experiments were performed with male wild-type mice (C57Bl/6J line) and were bred at Charles River Laboratories (L'Arbresle, France). All mice were transported to our facilities at eight weeks of age, housed under a 12h light-dark cycle with ad libitum access to food and water.

CX3CR1-GFP^+/−^ mice [33] were kindly provided by the *Unité d'Histopathologie humaine et modeles animaux* of the Institut Pasteur in Paris, France.

The experiments described in the present work were conducted in accordance with the guidelines on the ethical use of animals from the European Community Council Directive of 24 November 1986 (86/609/EEC) and in accordance with institutional animal welfare guidelines and were approved by the CETEA Ethics committee, protocol number 2013-0056 Animalerie Centrale and Médecine du Travail, Institut Pasteur.

### Stereotaxic injections and chronic cranial window

Twelve week old mice were anesthetized with ketamine (Imalgen 1000, 10% in PBS; Rhone Mérieux) and xylazine (Rompun, 2% in PBS; Bayer AG), 10 ml/kg i.p. The stereotaxic injections and chronic cranial windows were performed as previously described [39, 55]. Briefly, the skull was carefully thinned using a dental drill over the region of interest and the thinned bone was removed using forceps, leaving the dura intact. 200 nl of GCaMP6f expressing Serotype 2.1 AAV virus under the synapsin-1 promoter (AAV.syn. GCaMP6f.WPRE.SV40, 2.2e13 GC/ml, University of Pennsylvania Vector Core, catalog number; AV-1-PV2822, lot; CS0261WL) was injected bilaterally at the following coordinates into PrLC: AP, +2.8 mm from bregma; L, ±0.5 mm; and DV, −0.3 to −0.1 mm from the skull using a Nanoject II^TM^ (Drummond Scientific) at the slow infusion setting. For WT control mice, 2 μl of AAV1.CAG. tdTomato.WPRE.SV40 1.52e13 GC/ml (University of Pennsylvania Vector Core, catalog number; AV-1-PV2126) diluted in PBS 1X, was injected bilaterally at the same coordinates as described above. For the WT-hAPP mice, 2 μl of AAV-EF1a- hAPP-SLA-FLAG (2,2e12 GC/ml.) was also injected bilaterally at the same coordinates. The glass pipette was left in situ for an additional 5 min before being slowly withdrawn. The cranial window was covered with a circular coverglass (5mm diameter) and was sealed to the skull with dental cement (Coffret SUPERBOND complet, Phymep).

### Immunofluorescent staining

Mice were deeply anesthetized with a lethal dose of ketamine/xylazine before intracardiac perfusion with ice cold PBS, followed by 4% PFA (Sigma-Aldrich, Saint Louis, MO, USA). The brains were removed and post-fixed by immersion in 4% PFA for 2 days at 4°C. The brains were then immersed in 30% sucrose in PBS overnight at 4°C for cryoprotection. Serial 40 μm coronal sections were cut using a sliding microtome (Leica Microsystems) and transferred to PBS. Slices were incubated in 10% normal goat serum (NGS) and 0.2% Triton X- 100 in PBS for one hour, then washed in PBS and incubated with various combinations of primary antibodies: rabbit anti-GFP (1:2000; Life Technologies, Invitrogen, Carlsbad, CA, USA), rabbit anti-GFAP (1:1000; Chemicon, AB5804, Temecula, CA), mouse anti-VHH V31-1 (1:500; kindly provided by Pierre Lafaye [25]) and mouse anti-FLAG (1:1000; Sigma Life Science, F1804, France). A mouse anti-His antibody (1:1500; Sigma- Aldrich, Saint Louis, MO, USA) was used for amplifying the VHH signal. Fluorophore-conjugated secondary antibodies were used with cy3-anti-mouse and Alexa 488-anti-rabbit (Life technologies, Eugene, OR, USA) at a dilution of 1:500 for 3 hours at RT. After DAPI (Sigma-Aldrich, Saint Louis, MO, USA) incubation, the slices were mounted on slides ProLong Gold Antifade Reagent mounting medium (Life Technologies, Molecular Probes, Carlsbad, CA, USA). Images were acquired with a Zeiss epifluorescent microscope and a confocal microscope (Zeiss LSM 700, Heidelberg, Germany).

### Immunohistochemistry

40 μm coronal sections were incubated for 30 min in NH4Cl 50 mM, Lysine 1 mM and Glycine 1 mM in PBS, then one hour in 3% H_2_O_2_ in PBS for neutralisation of endogenous aldehydes and peroxidases, respectively. Slices were incubated in 10% NGS and 0.2% Triton X- 100 in PBS (Tx-PBS) for 1 hour and then with the primary biotinylated antibody, mouse anti-4G8 (1:500; Covance, Dedham, MA, lot: 09EC00860) or AT100 (1:100; Thermo Scientific, MN 1060, lot: QE202937), diluted in 2% NGS and 0.2% Triton X-100 in PBS and incubated overnight at 4°C. Slices were rinsed with Tx-PBS and incubated in ABC mixture for 30 min with gentle shaking (Elite ABC, PK-6100, Vector Laboratories, Burlingane, CA). After 3 washes with Tx-PBS, the sections were developed in DAB solution (Vector Novared substrate kit, SK-4800, Vector Laboratories, Burlingane, CA) for 5-10 minutes. Finally, slices were washed with distilled H_2_O and mounted in aqueous mounting media. Amyloid plaques and NFTs were visualized with light microscopy.

### Quantitative analysis of immunofluorescent images

The analysis of the Aβ diffusion, *in vivo* microglia images and the immunolabeling of astrocytes was performed using Fiji (ImageJ, NIH). For the microglia, 1024x1024 resolution images (163.225 × 163.225 um) were analyzed for both sham operated and AAV-hAPP injected mice. The same settings (laser power and gain) were used during the acquisition of the images. First, we set the scale in μm by adding the distance in pixels. For the quantification of cell area (somas and processes) the perimeter of the cells’ process tips was manually marked using the polygon selection tool, whereas for the soma area, we manually traced the somas of the cells within the whole field. In the set measurements menu we selected “area and then measure”. For the Aβ diffusion 5 slices were analyzed from 3 control mice and 5 slices from 3 AAV-hAPP-SLA injected mice. For the microglia analysis, 10 *in vivo* two-photon images from 3 control mice and 8 *in vivo* two-photon images from 3 AAV-hAPP injected mice were analyzed, n = 150 cells. For the analysis of astrocytic density between sham and AAV-hAPP injected mice, the rectangle tool was used to select a region of interest (ROI) of 200 × 200 um. The same size of ROI was selected for both sham and hAPP brain slices in the injection area. A ROI was also drawn in an area without fluorescence to be used for background subtraction. The net average fluorescence intensity in the ROI was calculated for the different groups and the average intensity values of AAV- hAPP injected mice were normalized by values obtained in sham mice. 6 slices were analyzed from 3 control mice and 6 slices from 3 AAV-hAPP-SLA injected mice. For these experiments, all parameters during image acquisition were kept identical.

### Quantitative analysis of immunohistochemical labeling

The analysis of optical densities for the immuno-histochemical labeling of amyloid plaques and neurofibrillary tangles was also performed in Fiji. The images first underwent color deconvolution. The H DAB setting was selected as the labeling method from the vectors and the analysis was performed and measurements were set to the mean grey value. ROIs were selected (the prelimbic cortex area was validated by taking mosaics of each brain slice), always maintaining the same size of box for both sham and hAPP brain slices. Optical density numbers were acquired using the formula OD = log (max intensity/mean intensity). The values were normalized by values obtained in sham mice. For the quantification of amyloid plaques, 8 slices were analyzed from 4 control mice and 6 slices from 3 AAV-hAPP injected mice, whereas for the quantification of NFTs, 6 slices were analyzed from 3 control mice and 6 slices from 3 AAV-hAPP injected mice.

### In vivo two-photon imaging

*In vivo* imaging was performed with an Ultima IV two-photon laser-scanning microscope system (Bruker), using a 16 × 0.8 NA water immersion objective (Nikon) with a femtosecond laser (MaiTai DeepSee, Spectra Physics, Mountain View, CA, USA) tuned to 950 nm for imaging of GCaMP6f expressing cells. Time-series movies of neuronal populations expressing GCaMP6f were acquired at 7 Hz (182 × 182 microns). Each focal plane movie duration was 3.6 minutes (1500 frames) to track spontaneous neuronal activity. Care was taken to use less than 10 mW of laser power at the surface of the tissue. For *in vivo* two-photon imaging of microglia cells of the CX3CR1 mice (also injected with the AAV1.CAG.tdTomato vector), the femtosecond laser tuned to 960 nm and laser power was kept below 5 mW to avoid phototoxic effects. Time series were acquired (1024x1024 pixels) at a 10-second interval for a total of 10 min (60 iterations).

### Two-photon data analysis

Image analysis (also for immunostained images) was performed off-line with ImageJ software. The time series were registered using the “Image Stabilizer” plugin (K. Li, http://www.cs.cmu.edu/∼kangli/code/Image_Stabilizer.html). Regions of interest (ROIs) were manually selected in FIJI and processing of Ca^2+^ transients of individual neurons was performed automatically by using a custom-written toolbox in MATLAB (Mathworks) based on a previously published method [56]. A baseline correction algorithm was used in order to remove the slow time scale (< 0.05 Hz) changes in the fluorescence as previously described [56]. Based on the fact that action potential firing causes calcium influx into the cytoplasm via the opening of voltage-gated calcium channels and therefore one calcium transient in not necessarily translated to one action potential, we deconvolved spontaneous Ca^2+^ transients with a putative unitary (spike-evoked) event in order to estimate neuronal firing rates. The analysis of synchronously firing neuronal populations was performed as previously described [39].

### Code availability

The custom-written toolbox in MATLAB (Mathworks, 2014b) is available upon request.

### Statistical analysis

Data are presented as ± SEM. The *P* values were obtained by a two-tail Students's t test comparing control and APP injected groups’ images. Kruskal-Wallis one-way analysis of variance combined with multiple comparison testing was applied on the activities (transients/min and spikes/min) of the neurons in all mouse groups in order to study the statistical similarities. We used Welch's Test ANOVA as a complementary test for heteroscedasticity. This test gave similar results as the Kruskal-Wallis test with the same level of significance.

## SUPPLEMENTARY MATERIAL








