# Supplementary material for: Early and progressive deficit of neuronal activity patterns in a model of local amyloid pathology in mouse prefrontal cortex
Source: Aging (Albany NY). 2016 Dec 20;8(12):3430–49. doi: 10.18632/aging.101136 (PMC5270678; doi:10.18632/aging.101136)
Supplement: Supplementary file 1 [file aging-08-3430-s001.pdf]

SUPPLEMENTARY MATERIAL

Please browse the links in Full Text version of this manuscript to see Supplementary Movies.

Supplementary Movie 1.

In vivo two-photon movie of a 3D projected z-stack of PFC in a CX3CR1-GFP$^{+/-}$ mouse. Microglia cells are shown in green. The red represents AAV1.CAG.tdTomato infected cells. The z-stack projected in 3D using Imaris software (Bitplane, Zurich, Switzerland).

Supplementary Movie 2.

In vivo two-photon time-series of microglia cells in the PFC of a CX3CR1-GFP$^{+/-}$ mouse (sham not injected with the AAV-hAPP-SLA). Resting state microglia cells characterized by a small cell body and highly elaborated thin processes.

Supplementary Movie 3.

In vivo two-photon time-series of microglial cells in the PFC of a CX3CR1-GFP$^{+/-}$ mouse injected with the AAV-hAPP-SLA. Microglia are characterized by an ameboid form, a feature of microglial activation.

Supplemental file. Related paper in press.
